# Development of Approaches and Metrics to Measure the Impact and Improve the Clinical Outcomes of Patients With Frailty in the Era of COVID-19. The COMETA Italian Protocol

**DOI:** 10.3389/fonc.2022.828660

**Published:** 2022-06-02

**Authors:** Nicola Silvestris, Valeria Belleudi, Antonio Addis, Fulvia Pimpinelli, Aldo Morrone, Salvatore Sciacchitano, Rita Mancini, Vito Michele Garrisi, Massimo Costantini, Gennaro Ciliberto, Vincenza Frisardi, Giulia Piaggio

**Affiliations:** ^1^Istituto Tumori “Giovanni Paolo II” of Bari, IRCCS, Bari, Italy; ^2^Department of Biomedical Sciences and Human Oncology, University of Bari “Aldo Moro”, Bari, Italy; ^3^Department of Epidemiology, Lazio Regional Health Service, Roma, Italy; ^4^San Gallicano Dermatological Institute, IRCCS, Roma, Italy; ^5^Azienda Ospedaliera Universitaria Sant’Andrea, Sapienza University, Roma, Italy; ^6^Geriatric Unit, Azienda Unità Sanitaria Locale (AUSL), IRCCS, Reggio Emilia, Italy; ^7^IRCCS, National Cancer Institute Regina Elena, Roma, Italy

**Keywords:** pandemic, SARS-CoV-2, cancer screenings, tracking fragile patients, blood biobank, immune response

## Abstract

The outbreak of the coronavirus 2 disease 2019 (COVID-19) puts an enormous burden on healthcare systems worldwide. This may worsen outcomes in patients with severe chronic diseases such as cancer, autoimmune diseases, and immune deficiencies. In this critical situation, only a few available data exist, which do not allow us to provide practical guides for the treatment of oncological or immunocompromised patients. Therefore, a further step forward is needed, addressing the specific needs and demands of frail patients in the pandemic era. Here we aim to present a protocol of a study approved by an ethical committee named “CO.M.E.TA”. CO.M.E.TA protocol is a network project involving six Italian institutions and its goals are: i) to measure and compare the impact of the pandemic on the access of cancer and immunocompromised patients to therapies in three Italian regions; ii) to assess how reorganizational measures put in place in these different institutions have impacted specific metrics of performance; iii) to establish a COVID-19 Biobank of biological samples from SARS-CoV-2 infected patients to be used to study immunological alterations in patients with immune frailty.

## Introduction

The COVID-19 pandemic started in December 2019 in Wuhan, the Chinese province of Hubei. More than 158,000 COVID-19 patients have died in Italy to date (https://www.epicentro.iss.it/coronavirus/sars-cov-2-sorveglianza-dati). Compared to the period of the first epidemic wave (March to May 2020), in the period of the second epidemic wave (October 2020 to July 2021), deceased people have a more significant clinical complexity, as demonstrated by the higher number of comorbidities. Overall, only 2.9% of patient deaths presented with no comorbidities, while 11.5% with a single comorbidity, 18.1% with two, and 67.4% with three or more. Beyond the direct effects of the COVID-19 pandemic in terms of morbidity and mortality, a wide range of indirect impacts on the health status of the population is expected. The need to allocate significant amounts of healthcare resources to the COVID-19 emergency, deferral of routine healthcare visits, and invitation to avoid medical controls, if not strictly necessary, may have led to interruptions of disease management undersupply of chronic treatments. This may result in decreased adherence to chronic drug treatment and cancellations of scheduled lab tests or exams. Consequently, the health status of patients with chronic pathologic conditions may have worsened during and beyond the crisis. Here we present a protocol that entails the collaboration of a network of six Italian institutions with complementary expertise in order to conduct an analysis of these issues.

Patients with cancer, autoimmune disease, and immune deficiencies represent populations with varying immunocompetence, which may translate into higher susceptibility to SARS-CoV-2 and, for this reason, we defined them as frail populations. In cancer patients, recent surgical procedures and/or radiotherapy, and/or systemic treatments could increase the risk of infections due to hematologic toxicity. Targeted therapies may interfere with the innate and adaptive immune system (e.g., tyrosine kinase inhibitors), while immune-checkpoint inhibitors may worsen the course of COVID-19. Similarly, in chronic pathologic conditions with underlying autoimmune mechanisms, such as psoriasis and bullous diseases, and patients with immunodeficiencies (e.g., HIV-infected persons), the underlying pathogenic mechanisms and treatments in the course may impact on COVID-19 outcome (1–9). Although cancer and immune disease may be risk factors for the emergence and evolution of COVID-19, prospective epidemiological data are needed to determine how many Italian frail patients have been infected with the SARS-CoV-2 virus as well.

**Figure 1:**
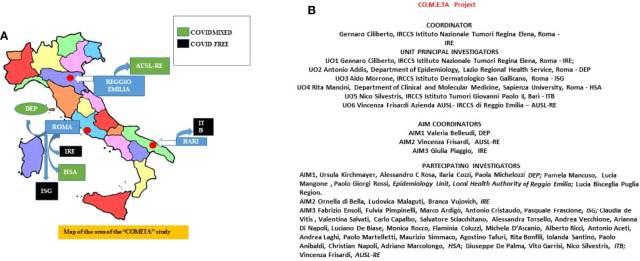
**(A)** Schematic geographical representation of the six sites involved in the protocol. In the blue light, boxes are indicated the names of the involved towns. The names of the involved sites are indicated in the green and black boxes. The green box indicates COVID-19 free sites, and black boxes indicate COVID-19 mixed sites. **(B)** Organogram of the people involved in the different activities of the study protocol.

In this regard we plan collection of data from five healthcare centers located in three geographical areas in Italy, namely AUSL-IRCCS di Reggio Emilia (AUSL-RE) in Emilia-Romagna region representative for North; IRCCS Istituto Nazionale Tumori Regina Elena, Roma (IRE), IRCCS Istituto Dermatologico San Gallicano, Roma (ISG), and Department of Clinical and Molecular Medicine, Sapienza University, Roma (HSA) in Lazio region representative for Center; IRCCS Istituto Tumori Giovanni Paolo II, Bari (ITB) in Puglia region representative for South Italy, respectively. In addition, we collect data from the Department of Epidemiology of the Lazio Regional Health Service (DEP). The organogram of the protocol participating centers and involving researchers is schematized in **Figures 1A, B**.

## Challenges

Recent studies indicate that cancer patients with COVID-19 experience severe outcomes, with a lethality rate ranging from 13% to 33%, compared to 0.5% to 2% in the general population (10). In Italy, in July 2021, the estimated number of deaths attributable to COVID-19 who presented active cancer in the previous 5 years was 16.3%, while 4.6% showed a positive anamnesis for autoimmune disease (https://www.epicentro.iss.it/coronavirus/bollettino/Bollettino-sorveglianza-integrata-COVID-19_21-luglio-2021.pdf).

It is well known that the development of cancer itself, as well as surgical procedures, radiotherapy, and/or systemic treatments, can lead to immune suppression (11), thus increasing the risk of SARS-CoV-2 infection (1, 12–14). On the other side, COVID-19 negatively impacts a broad spectrum of cancers (4, 15–17).

Considering the interaction between SARS-CoV-2 and the host immune system, patients with severe immunological diseases may be at higher risk of an aberrant immune response in the case of SARS-CoV-2 infection and may therefore require additional precautions (18).

Since March 2021, frail people have been enrolled in the Italian anti-COVID-19 vaccination program. The data suggest that some solid and hematologic cancer patients exposed to therapies develop relatively lower antibody titers after vaccination. This underscores the need for dynamic serological monitoring upon vaccination as well as specific safeguard strategies to reduce their risk of re-infection in view of relaxing mandates, such as mask mandatory and social distancing (19–22).

Apart from the impact of COVID-19 and the limitations of vaccine responses in frail patients, another challenge during the COVID-19 crisis’s emergency phases is that patients with cancer and immune system disorders may have experienced limitations in seeking healthcare and receiving pharmacological treatments. Analysis of both the direct and indirect impact of the COVID-19 pandemic in patients with immune-mediated inflammatory diseases in Italy showed a higher risk of SARS-CoV-2 infection and lower use of biologic drugs (23).

All these elements together may exacerbate the potential negative health consequences of the COVID-19 crisis in these populations (4, 24).

In order to meet these challenges, the centers involved in the protocol mentioned earlier, both COVID-19 and non-COVID-19 centers, have recently undergone a radical and incredibly rapid reorganization of the management of their frail patients aimed to preserve elective surgery and urgent treatments and to postpone only routine controls (18, 25–29).

The results coming from this protocol will propel the field of COVID-19 impact on particularly vulnerable categories of patients. The findings of this study will aid in determining the conditions under which healthcare organizations must operate in the event of a pandemic in order to protect patient’s rights to care. This protocol also will lead to the development of intervention strategies and guidelines to facilitate decision-making and to anticipate new and emerging healthcare needs.

## Objectives

**Figure 2:**
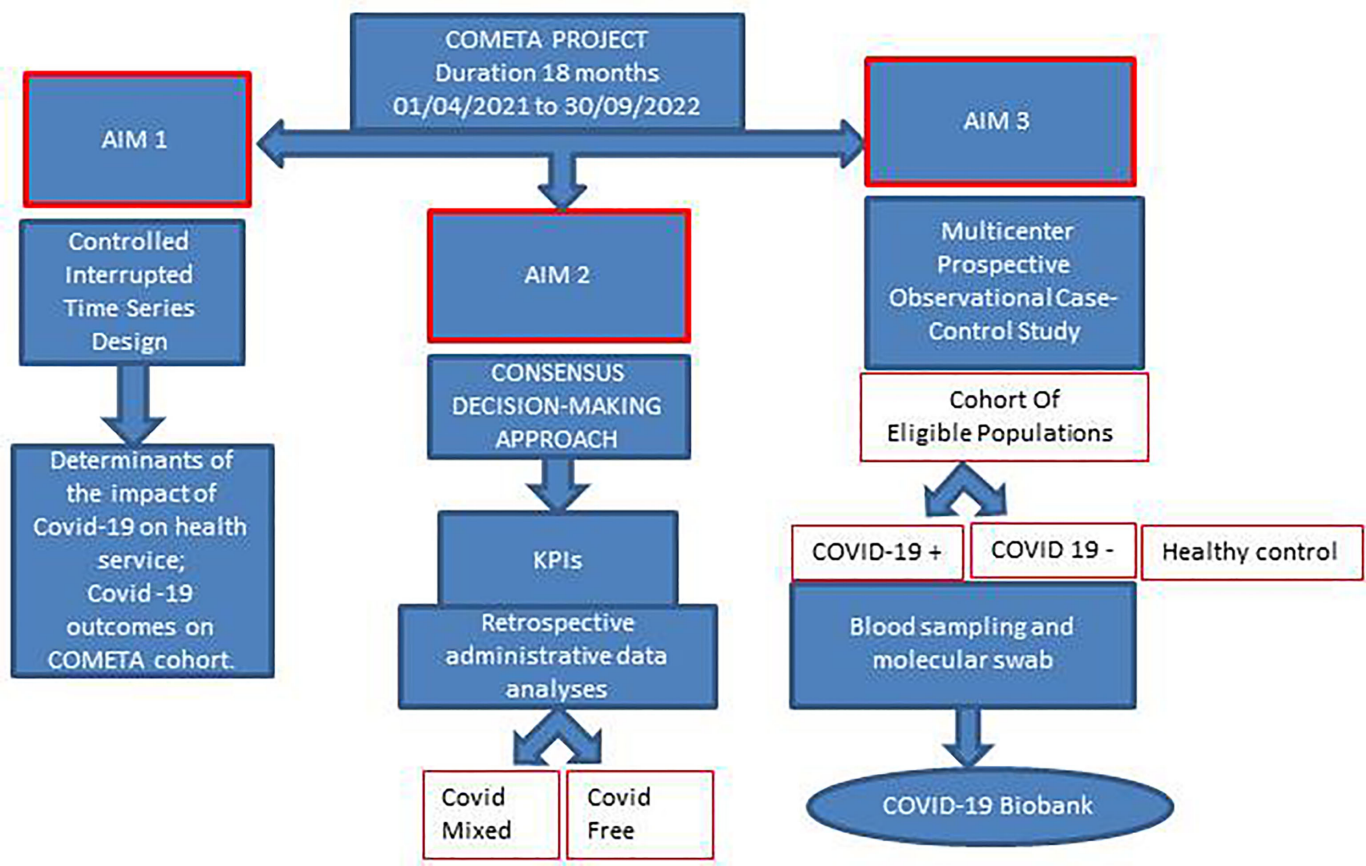
Flow chart of the entire project.

The CO.M.E.TA protocol (acronym of COvid/Migliorare/Esiti/fragiliTA’ that means to ameliorate the outcome of frail people in the COVID era) describes a network project involving six Italian operative units (OU), five hospitals, and one department of epidemiology. This project is expected to have a national impact as the OUs are located in three Italian regions, Lazio, Emilia-Romagna, and Puglia being located in the north, center, and south of Italy, respectively. Assuming that the COVID-19 pandemic had an effect on treatment adherence and follow-up of frail patients in the past and will continue to do so in the future, with long-term negative consequences for chronic disease outcomes, we believe that *ad hoc* interventions can significantly improve the quality of healthcare services provided to frail patients in both COVID-19- and COVID-19-free hospitals. In addition, it seems relevant that collecting biological material from frail patients, with or without COVID-19, today may be of extreme utility in the future to study different clinical outcomes through specific biological alterations such as acquired or innate immune response or to identify/validate circulating biomarkers. The CO.M.E.TA protocol fits precisely into this context and its objectives are: i) to assess the direct and indirect impact of the COVID-19 epidemic on cancer and immunocompromised patients in terms of adherence to treatment protocols and severity of chronic morbidity outcomes in the three Italian regions; ii) to measure implementation of the non-COVID status in non-COVID centers (i.e., implementation of telemedicine tools with “user friendly” interfaces on multimedia communication) and the differential hospital access strategies in mixed-mode centers and how the reorganizational measures put in place in the three hospitals have impacted specific metrics of performance; iii) carry out a serial collection and storage in a dedicated bank of biological samples such as whole blood, serum, plasma, and peripheral blood mononuclear cells (PBMCs), to be used to investigate the serum levels of anti-SARS-CoV-2 antibodies (IgG/IgM/IgA), specific biological alterations such as acquired or innate immune response, and the presence of specific circulating biomarkers. A schematic flowchart of the entire project is presented in **Figure 2**.

## Study Design

### General Schema

The current protocol describes a multicenter prospective observational case-control study to develop approaches and metrics for assessing the impact of SARS-CoV-2 infection on frail patients at various disease/therapy stages in order to improve frail patient clinical management in the COVID-19 era. Moreover, the protocol describes the generation of a biological bank of oro-pharyngeal swabs and blood samples.

### Study Duration and Enrollment

The study duration will last 18 months. As the COVD-19 pandemic trend is not forecastable, we assume to enroll almost 200 patients. Recruitment is expected to end September 30, 2022, and in any case, when the pandemic ends.

### Participating Sites

As described above, the network involves five healthcare centers, IRCCS Istituto Nazionale Tumori Regina Elena (IRE), IRCCS Istituto Dermatologico San Gallicano (ISG), Department of Clinical and Molecular Medicine, Sapienza University (HAS), IRCCS Istituto Tumori Giovanni Paolo II (ITB), Azienda AUSL-IRCCS di Reggio Emilia (AUSL-RE), both non-COVID and mixed-mode institutions located in different geographical areas of Italy (**Figures 1A, B**). In addition, we collect data from the Department of Epidemiology, Lazio Regional Health Service (DEP). The protocol has been approved by the ethics committees of all participating institutions

### Compliance Statement

This study will be conducted in full accordance with all applicable Research Policies and Procedures and all applicable regulations (Helsinki Declaration, the principles of Good Clinical Practice, and all the requirements of regulatory authorities and key European and national regulations). The investigators will perform the study according to this protocol, obtain consent and assent, and report unanticipated problems involving risks to subjects or others. The data and documentation produced by the activities described in this protocol will be stored following national laws/regulations to ensure confidentiality.

## Participants and Inclusion/Exclusion Criteria

### Patients’ Cohort

The CO.ME.TA protocol involves the consecutive enrolment of frail patients at different stages of the disease and undergoing various treatments. Precisely, the protocol was planned to enroll patients with cancer disease, among them hematological cancer patients that could undergo transplantation, as well as patients with autoimmune disorders and HIV infection. The stratification of the different cohorts will be done retrospectively. In the case of future research and/or validation studies using collected biological samples, the different cohorts will be identified each time according to the research question that the study wants to address that the survey seeks to answer, and *ad hoc* projects will be prepared.

### Control Cohort (Healthy Subjects)

We will use the saved samples in the biobank to validate projects. It is necessary to provide an additional control cohort of healthy subjects compared with the patient cohorts defined above. For this purpose, the protocol includes biobanking blood samples from a cohort of healthy donors (who meet the requirements of suitability for blood and blood components), available at the IRE transfusional center. The donators would also have an absence of sepsis prior to the last three months, no previous neoplasms, lack of concomitant autoimmune pathologies, absence of chronic pathologies, and no concomitant drug use. As for the patients, the protocol states that all healthy subjects enrolled in the study will write an informed consent for participation in the study.

### Inclusion Criteria

- Age > 18 years.
- Written informed consent for participation in the study.

The protocol foresees that i) in the period between April 1, 2021, and September 31, 2022, all COVID-19 positive frail patients identified by molecular swabs taken at participating institutions will be included in the study; ii) on a quarterly basis, a cohort of equal numbers of COVID-19 negative frail patients will be identified and paired by disease (where possible by disease/therapy setting), gender, and age.

### Exclusion Criteria

Given the pragmatic and innovative approach of the studies reported in this protocol, no exclusion criteria are identified.

## Data Collection, Sample Size, and Statistical Analysis

A precise procedure for the medical data collection of enrolled patients is planned in the current protocol. Data collected as part of this study will be entered and stored using REDCap (Research Electronic Data Capture) database. REDCap is a secure web-based software solution and workflow methodology supporting clinical and translational research databases. REDCap will be developed by the informatics core at IRE and shared with the Principal Investigators of each involved center. The database will incorporate range checks and between-variables consistency checks to ensure quality control. The system will signal the presence of questionable or potentially incorrect items. The database will be password-protected, stored, and backed up on a daily basis by IRE. Access to the directory where the data will be stored will be restricted to the primary research team. Computer files will only contain study identifier codes and will be password-protected, with access limited to members of the research team.

**Figure 3:**
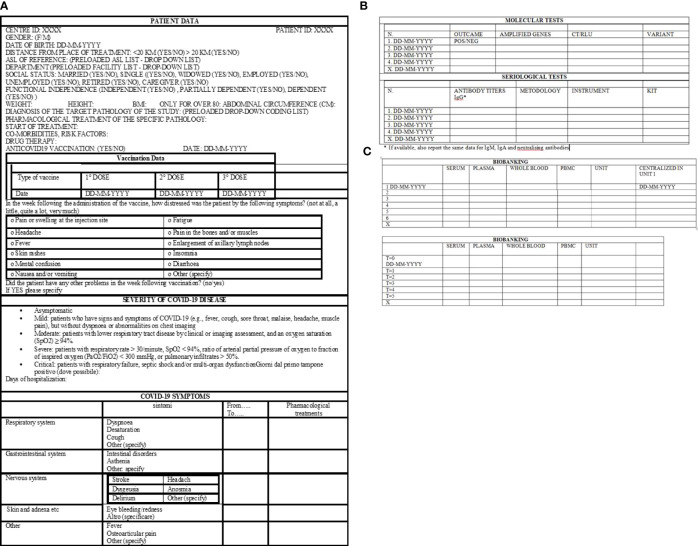
Standard electronic case report form, eCRF. **(A)** Demographic information, type of vaccination, COVID-19 disease history. **(B)** Information about molecular and serological tests. **(C)** Data about sample biobanking.

A standard electronic case report form (e-CRF) is designed and developed by the coordinator Institute, IRE, and it is available to all other centers. Following registration, patient data will be collected in the e-CRF. Upon ID and password, the project manager of each institute has the possibility to access the eCRF, enroll its patients, and enter all required features. The protocol plans to collect demographic information, disease history, COVID-19 symptomatology (**Figure 3A**), COVID-19 molecular test details, COVID-19 serological test results (**Figure 3B**), and biobanking informatics (**Figure 3C**). In addition, clinical analyses (**Supplementary Table 1**), anamnestic data, and pharmacological treatment pieces of information (**Supplementary Table 2**) will be collected.

The number of subjects to be included in this project will depend on the number of recruited patients who will result in COVID-19 positive during the study period.

For each disease, the same number of patients negative for COVID-19 disease and healthy controls will be included. Based on the achieved sample size, the possibility of performing statistical tests to highlight differences between groups will be evaluated.

The data will be analyzed by the biostatisticians identified in each center. Depending on the number of enrolled patients, *ad hoc* projects will be drawn up, describing the objectives to be reached and the specific methodology.

All data and records generated during this study will be kept confidential following institutional policies and subject privacy laws. The investigators and other site personnel will not use such data and documents for any purpose other than conducting the study.

## Procedures

**Figure 4:**
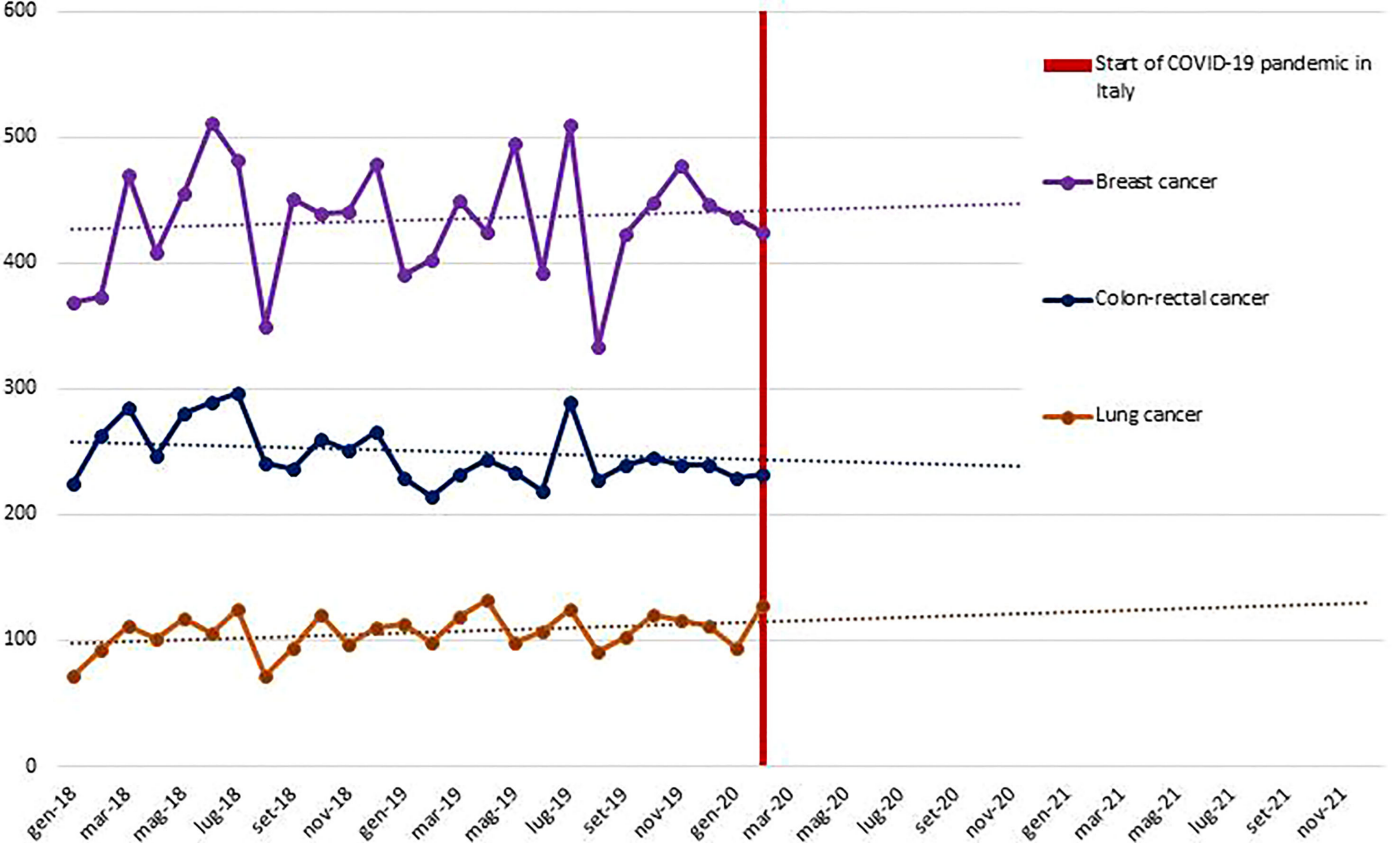
Volumes of incident surgical interventions for breast, colon-rectal, and lung malignant cancer during the pre-COVID19 pandemic and expected trend lines - Lazio Region.

*Aim 1*. Data on anamnestic examination, evaluation, and patient care are collected from the COVID-19 registry and health information systems made available through participation of the DEP in the Lazio Regional Health Service network. Moreover, thanks to the collaboration of DEP with the epidemiology departments of other regions (i.e., Emilia, Puglia) the data are representative of different areas of Italy. The planned statistical analysis will identify the determinants of the impact of COVID-19 on health service uptake by applying a controlled interrupted time-series design. An example of analysis in the oncological area is depicted in **Figure 4** and shows volumes of incident surgical intervention pre-COVID-19, potential variations from expected values will be studied. Particular attention is given to predictors of severity of COVID-19 outcomes in the study population. The impact of COVID-19 on indicators of medication compliance or switching to other therapies will be investigated. As an example, in **Table 1**, we report background rates (years 2018 to 2019) of several indicators that will be considered in the oncological area. In the CO.M.E.TA protocol, we propose comparing these background rates with those observed in the pandemic. In the subsets of frail patients, for whom short-term outcomes can be predicted, a comparison of the study cohorts (COVID-19 and non-COVID-19 frail patients) will give us the opportunity to assess the impact of the COVID-19 crisis on disease-specific (progression-free survival, objective response, clinical benefit) and non-disease specific (access to intensive care unit) endpoints.

**Table 1:** Indicators (monthly *%*) of neoadjuvant and adjuvant treatment defined using health information systems: background rate for breast, colon-rectal, and lung in the Lazio Region.

	Breast	Colon-rectal	Lung
	monthly rats 2013-19	monthly rate 2013-19	monthly rats2013-19
Patients with chemotherapy in the 60 days pre admission	3.9%	1.7%	1.3%
Patients with radiotherapy in the 60 days pre admission	0.1%	3.0%	1.3%
Patients with chemotherapy in the 50 days post discharge*	18.7%	27.0%	19.3%
Patients with radiotherapy in the 60 days pre admission	12.8%	0.7%	*2.3%*
Patients with immunotherapy in the SO days post discharge	7.3%	0.2%	0.8%
Patients with hormone therapy in the 60 days post discharge	43.8%	**-**	**-**

*The following pre-and post- COVID-19 indicators will be analyzed for patients receiving chemotherapy:

■median time between first chemotherapy administration and discharge;

■pattern of drug use and formulations (oral, intravenous and subcutaneous);

■percentage of patients with first chemotherapy at the same hospital that performed surgery.

*Aim 2*. All activities put in place to protect frail patients during the first wave of the pandemic are continued and implemented. In particular, we aim to measure the impact of a) minimizing the number of hospital visits and admissions also by using telemedicine strategies; b) postponing non-urgent surgery; c) enhancing patient information; d) outsourcing to the territory all possible therapies with the administration of drugs at home; e) carrying out serological and virological screening of all hospitalized patients and of the staff involved in order to guarantee a non-COVID-19 status both in non-COVID centers and in non-COVID-19 areas of mixed-mode institutions. All these measures are expected to achieve the following results: 1) collect real data on the effects of new regimens on patients; 2) determine the incidence of symptomatic vs. asymptomatic subjects; 3) determine the morbidity of COVID-19 in frail patients.

To cope with the impact of the COVID-19 pandemic preserving the purpose and mission of the healthcare organizations, all activities have been reorganized to protect fragile patients.

In particular policies of a) minimizing the number of hospital visits and admissions also by using telemedicine strategies; b) postponing non-urgent surgery; c) enhancing patient information; d) outsourcing to the territory all possible therapies with the administration of drugs at home; e) carrying out serological and virological screening of all hospitalized patients and of the staff involved to guarantee a non-COVID-19 status both in non-COVID centers and in non-COVID-19 areas of mixed-mode institutions were implemented. The protocol presented here aims to measure the impact of all these measures to achieve the following results: 1) collect real data on the effects of new regimens on patients; 2) determine the incidence of symptomatic vs. asymptomatic subjects; 3) determine the morbidity of COVID-19 in frail patients.

**Figure 5:**
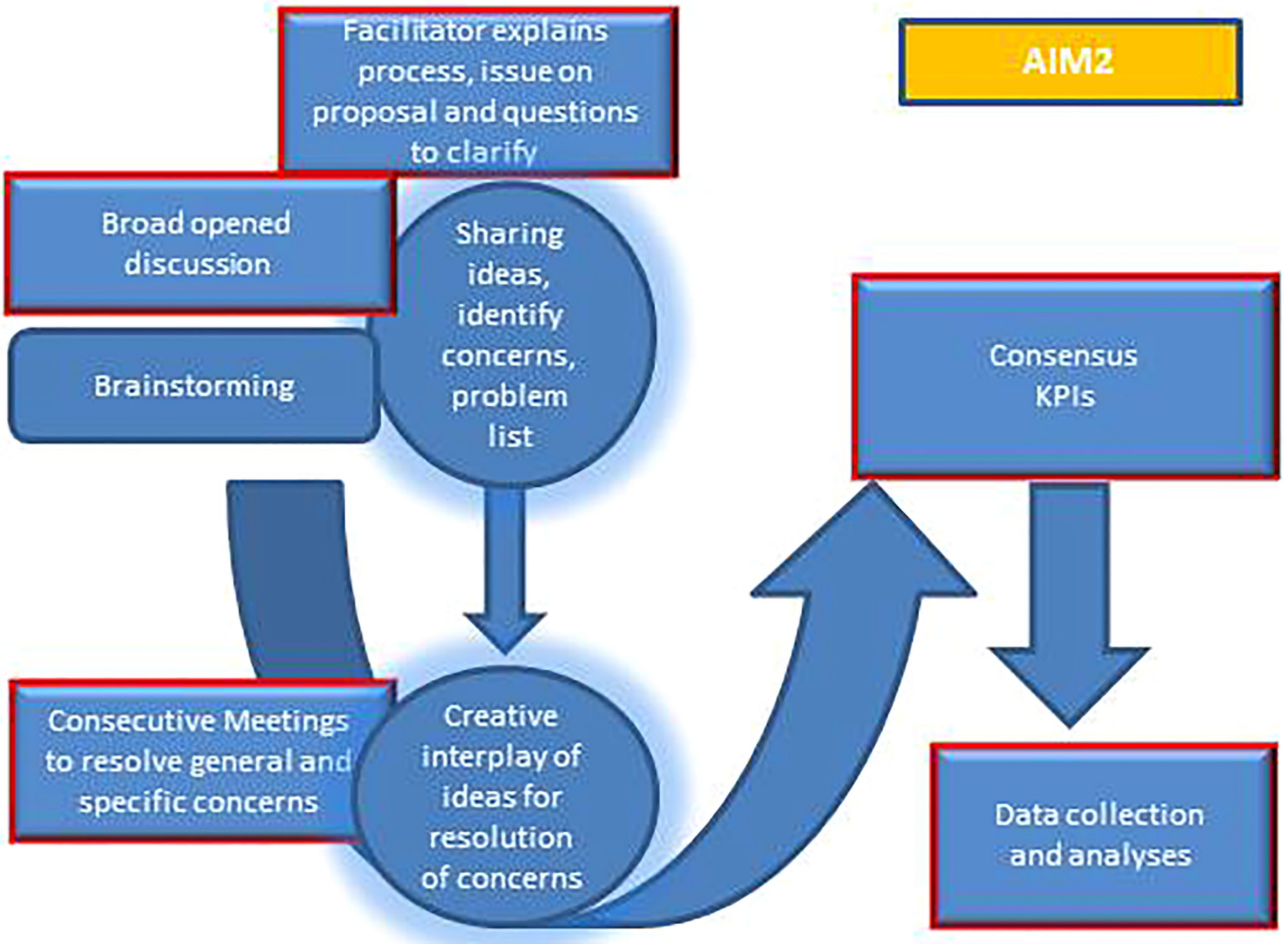
Flow chart of Aim 2.

The protocol strategy plans a consensus decision-making process (**Figure 5**). A panel of representatives of each institute involved in this project network will select key performance indicators (KPIs). KPIs will be simple or composite and will show how different modalities to face the COVID-19 pandemic (namely COVID and non-COVID hospitals) may affect healthcare organization performance to guarantee the quality of care in the clinical pathway of frail patients. KPIs for topical moments will be related to the oncologic volumes activities for the first appointment, treatment, and follow-up. A query of information flows, easily comparable amongst units involved in this project, will be applied with regard to the pre-pandemic (2019), pandemic (2020), and late pandemic (2022), divided by quarters. Regional differences, especially during the first COVID wave, were observed; therefore, the protocol aims to analyze data according to the Rt number, the index of Effective Reproduction Number. Using data from the administrative flows relating to the Operating Register, the hospital discharge databases (SDO), and the Single Regional Booking Centre, we will extrapolate data from the population target by ICD-10-CM codes (the international classification disease ten editions).

*Aim 3.* Recent evidence indicates that mutational variants of the virus can modulate the clinical course and spread of the disease (Grives et al., 2021). These crucial results emphasize the value of real-time generation and sharing of viral sequence data on a global scale, which has been one of the features of scientific efforts to combat the COVID-19 pandemic in 2020. Unfortunately, this practice is still very marginal in Italy. Of the sequences (whole or partial) deposited in GSAID to date, only 0.75% are sequences of the virus isolated from Italian patients (https://www.gisaid.org/).

In order to fill these gaps, the protocol CO.M.E.TA plans to establish a biobank of prospective samples from frail patients with or without COVID-19: a) biobanking of oro- and nasopharyngeal swabs for mutational profile analysis; b) biobanking of serum, plasma, whole blood, and peripheral mononuclear cells (PBMCs).

Concerning blood samples for the biobank, patient recruitment is carried out by all the participating centers, except for the DEP. IRE carries out donor recruitment.

**Figure 6:**
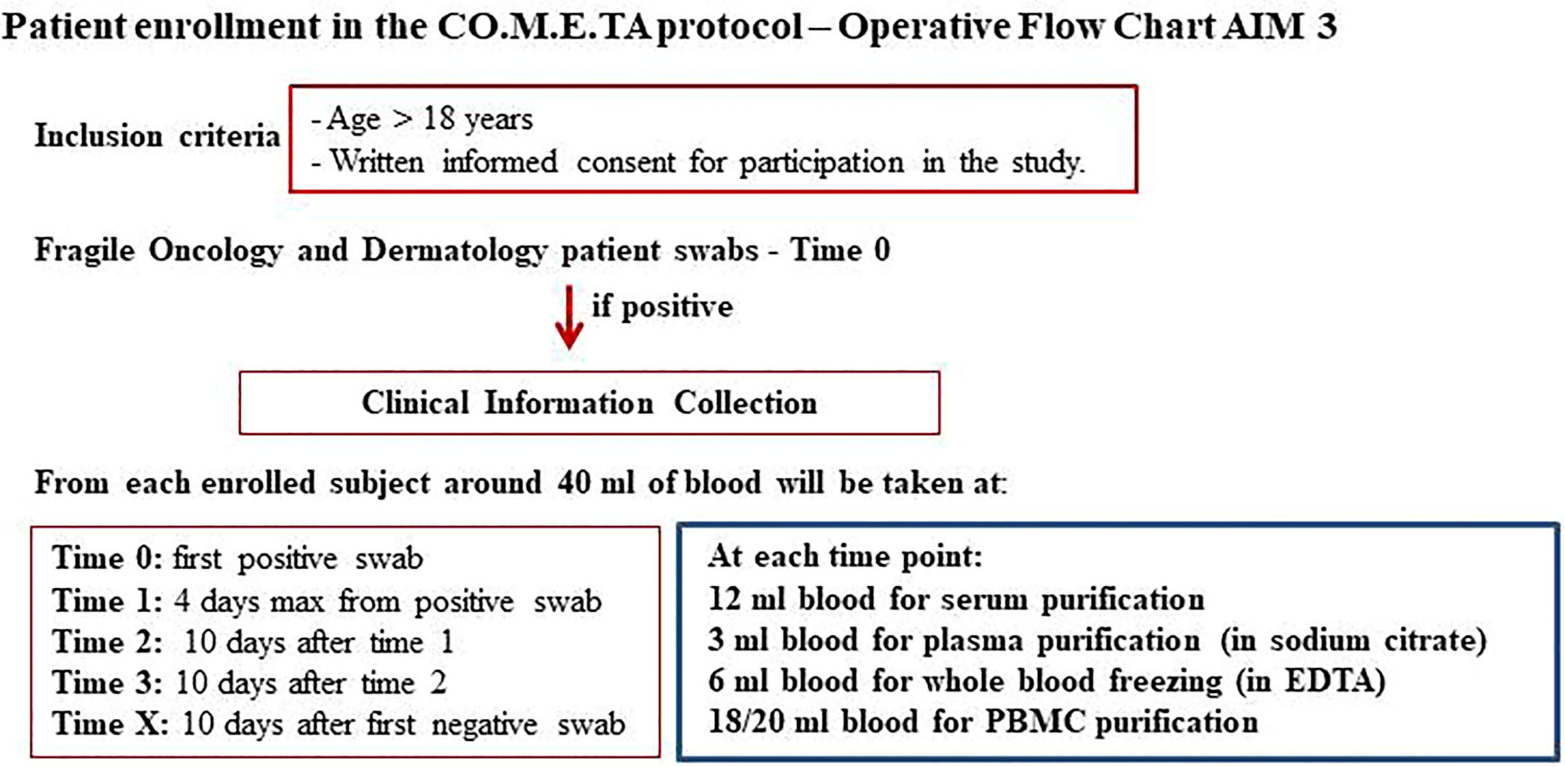
Flow chart of Aim 3. Strategy of infection assessment and time sampling shared with all participating sites.

HSA and AUSL-RE are mixed modality hospitals with COVID-19 sections where oncological patients are also admitted. At these hospitals, patients are enrolled in the protocol regardless of the stage of COVID-19 disease once their consent is obtained. Peripheral blood sampling of these patients and molecular and serological analyses are performed by hospital health workers at the time of the first positive swab result (T_0_), after 10 days (T_1_), after an additional every 10 days (T2, T3, etc.) until and the first negative swab result (TX) (**Figure 6**).

IRE, ISG, and ITB are COVID-19-free hospitals. These institutions have a dedicated channel to perform the nasopharyngeal swab for coronavirus outside the hospitals. The protocol plans to contact asymptomatic or paucisymptomatic SARS-CoV-2 positive subjects and enroll them in the study once they give informed consent. Health workers adequately equipped with safety devices go to the patient’s home to perform the blood sampling (T0). Ten days after the first positive swab, the patient returns to the hospital for a control swab. At this time, peripheral blood samples are taken in an external and protected route (T1). If the patient still tests positive, he/she usually returns after every 10 days, and further samples are taken at this time in the same way as described above (T2, T3, etc.). Once negative, a final sample is taken at the hospital laboratories (TX) (**Figure 6**).

The protocol plans that for each patient enrolled, only the first swab is biobanked. Approximately 40 ml of blood is collected from each subject at each experimental time: 6 ml of blood for serum purification, 3 ml of blood for plasma purification (in sodium citrate), 6 ml of blood for the whole blood-freezing (in EDTA), 18/20 ml blood for purification of PBMCs (**Figure 6**).

For the cohort of COVID-19-negative frail patients and the cohort of healthy donors, blood samples are collected only once (40 ml as described above), during access at participating centers. The samples are then centralized at the coordinator center, processed, and saved.

## Safety Management

Since the study procedures are not greater than minimal risk, adverse events reactions are not expected. If any unanticipated problems related to the research involving risks to subjects or others happen during this study, these will be reported.

## Patient Benefits of Study Participation

The research will be on voluntary participation, and it does not offer the prospect of direct medical or psychological benefit to the subjects, nor to others. There may be indirect benefits of reflecting on one’s own experience and contributing to scientific advances.

## Expected Outcomes

The network is highly complementary as it can be gathered from the range of expertise covering different branches of scientific research represented in the project. The network includes medical oncologist operative units (OU1, OU5, OU6), epidemiologists (OU2), immunologists (OU1, OU3, OU4), molecular pathologists (OU3, OU4), and molecular biologists (OU1 and OU4). The synergy resides in the fact that the network comprises hospitals and the Department of Epidemiology and Prevention of the Lazio Region, thus giving the opportunity to have access to a considerable amount of data.

In summary, the expected outcomes of this project are:

Aim 1) we expect to highlight the impact of COVID-19 and related diseases on frail patients, as evaluated in terms of social health difficulties caused by the COVID-19 outbreak.
Aim 2) we will show evidence of the impact of COVID-19 on drug adherence and medical services and on what are the best strategies adopted to maintain a high standard of care and patient safety.
Aim 3) we will establish sera and blood biobanks from SARS-CoV-2 infected patients, which can remain available to other institutions for future search for additional biomarkers. Unraveling the biological mechanisms associated with SARS-CoV-2 infection in frail patients is crucial to implementing strategies for early detection and protection of frail patients.

Ultimately our expected outcome is to develop general guidelines for clinical care of frail patients in the era of COVID-19, to be further evaluated by other institutions.

## Data Availability Statement

The original contributions presented in the study are included in the article/supplementary material. Further inquiries can be directed to the corresponding author.

## Ethics Statement

The studies involving human participants were reviewed and approved by Comitato Etico Sezione I.F.O. (Istituti Regina Elena e San Gallicano) – Fondazione Bietti - *Via* Elio Chianesi 53, 00144 Rome Italy. The patients/participants provided their written informed consent to participate in this study.

## Author Contributions

NS, VB, AA, FP, AM, RM, SS, VMG, MC, GC, VF and GP contributed to the conception and design of the study. NS, GC, VF and GP wrote the manuscript. GP prepared **Figures 1B** and **6**. VF prepared **Figures 1A**, **2**, and **5**, VB prepared **Figure 4** and **Table 1**. All authors contributed to the article with critical readings. NS and VB: these authors have contributed equally to this work and share the first authorship. VF and GP: these authors have contributed equally to this work and share the last authorship. All authors read and approved the final manuscript.

## Conflict of Interest

The authors declare that the research was conducted in the absence of any commercial or financial relationships that could be construed as a potential conflict of interest.

## Publisher’s Note

All claims expressed in this article are solely those of the authors and do not necessarily represent those of their affiliated organizations, or those of the publisher, the editors and the reviewers. Any product that may be evaluated in this article, or claim that may be made by its manufacturer, is not guaranteed or endorsed by the publisher.
