# Supplementary material for: Development of Approaches and Metrics to Measure the Impact and Improve the Clinical Outcomes of Patients With Frailty in the Era of COVID-19. The COMETA Italian Protocol
Source: Front Oncol. 2022 Jun 2;12:828660. doi: 10.3389/fonc.2022.828660 (PMC9215159; doi:10.3389/fonc.2022.828660)
Supplement: Supplementary file 1 [file Table_1.docx]

| **ANNEX 1 CLINICAL ANALYSES** | **DATE** |
| --- | --- |
| % Basophils | DD-MM-YYYY |
| % Eosinophils |  |
| % Lymphocytes |  |
| % Monocytes |  |
| % Neutrophils |  |
| albumin |  |
| ALT |  |
| AST |  |
| Azotemia |  |
| Total bilirubin |  |
| Creatinine clearance |  |
| Leukocyte count |  |
| Platelet count |  |
| Reticulocyte count |  |
| Creatinine |  |
| D-dimer |  |
| Serum electrophoresis |  |
| Haematocrit |  |
| Haemoglobin |  |
| Glycosylated haemoglobin |  |
| Erythrocytes |  |
| Serum erythroprotein (sEPO) |  |
| Ferritin |  |
| GGT |  |
| Blood glucose |  |
| Blood group |  |
| IL-1β |  |
| IL-6 |  |
| LDH |  |
| MCH |  |
| MCHC |  |
| MCV |  |
| Microalbumin |  |
| Na, K, Ca, Mg |  |
| PCR |  |
| Thyroid profile (FT3, FT4 etc.etc.) |  |
| RDW |  |
| Combs test |  |
| Circulating TNF a |  |
| Transferrin |  |
| ESR |  |
