# Supplementary material for: Development of Approaches and Metrics to Measure the Impact and Improve the Clinical Outcomes of Patients With Frailty in the Era of COVID-19. The COMETA Italian Protocol
Source: Front Oncol. 2022 Jun 2;12:828660. doi: 10.3389/fonc.2022.828660 (PMC9215159; doi:10.3389/fonc.2022.828660)
Supplement: Supplementary file 2 [file Table_2.docx]

| APPENDIX 2 - ANAMNESTIC DATA | Pharmacological Treatment | From |
| --- | --- | --- |
| Smoking behaviour |  | DD-MM-YYYY |
| Alcohol behaviour |  |  |
| Allergies |  |  |
| Arthritis and other rheumatic diseases |  |  |
| Chronic obstructive bronchitis |  |  |
| Cardiomyopathies |  |  |
| Diabetes |  |  |
| Arterial hypertension |  |  |
| Liver failure |  |  |
| Kidney failure |  |  |
| Dermatological diseases |  |  |
| Malnutrition and cachexia |  |  |
| Psoriasis |  |  |
| Transplants |  |  |
| Other diseases and conditions |  |  |
